# Effectiveness Assessment of Bispectral Index Monitoring Compared with Conventional Monitoring in General Anesthesia: A Systematic Review and Meta-Analysis

**DOI:** 10.1155/2024/5555481

**Published:** 2024-08-07

**Authors:** Yichun Gu, Jiajun Hao, Jiangna Wang, Peng Liang, Xinyi Peng, Xiaoxiao Qin, Yunwei Zhang, Da He

**Affiliations:** ^1^ Shanghai Health Development Research Center, Shanghai, China; ^2^ School of Public Health Zhejiang University School of Medicine, Hangzhou, Zhejiang, China; ^3^ Jiangxi University of Chinese Medicine, Nanchang, Jiangxi, China; ^4^ Department of Anesthesiology Day Surgery Center West China Hospital Sichuan University, Chengdu, Sichuan, China; ^5^ Department of Health Management School of Medicine and Health Management Tongji Medical College Huazhong University of Science and Technology, Wuhan, China

## Abstract

*Background and Objective*. The Bispectral Index (BIS) is utilized to guide the depth of anesthesia monitoring during surgical procedures. However, conflicting results regarding the benefits of BIS for depth of anesthesia monitoring have been reported in numerous studies. The purpose of this meta-analysis and systematic review was to assess the effectiveness of BIS for depth of anesthesia monitoring. *Search Methods*. A systematic search of Ovid-MEDLINE, Cochrane, and PubMed was conducted from inception to April 20, 2023. Clinical trial registers and grey literature were also searched, and reference lists of included studies, as well as related review articles, were manually reviewed. *Selection Criteria*. The inclusion criteria were randomized controlled trials without gender or age restrictions. The control groups used conventional monitoring, while the intervention groups utilized BIS monitoring. The exclusion criteria included duplicates, reviews, animal studies, unclear outcomes, and incomplete data. *Data Collection and Analysis*. Two independent reviewers screened the literature, extracted data, and assessed methodological quality, with analyses conducted using R 4.0 software. *Main Results*. Forty studies were included. In comparison to the conventional depth of anesthesia monitoring, BIS monitoring reduced the postoperative cognitive dysfunction risk (RR = 0.85, 95% CI: 0.73∼0.99, *P* = 0.04), shortened the eye-opening time (MD = −1.34, 95% CI: −2.06∼−0.61, *P* < 0.01), orientation recovery time (MD = −1.99, 95% CI: −3.62∼−0.36, *P* = 0.02), extubation time (MD = −2.54, 95% CI: −3.50∼−1.58, *P* < 0.01), and postanesthesia care unit stay time (MD = −7.11, 95% CI: −12.67∼−1.55, *P* = 0.01) and lowered the anesthesia drug dosage (SMD = −0.39, 95% CI: −0.63∼−0.15, *P* < 0.01). *Conclusion*. BIS can be used to effectively monitor the depth of anesthesia. Its use in general anesthesia enhances the effectiveness of both patient care and surgical procedures.

## 1. Introduction

Precisely assessing the depth of anesthesia remains a persistent challenge for clinical anesthesiologists. Conventional monitoring of anesthetic depth is primarily assessed by the patient's clinical signs and symptoms, such as changes in heart rate, blood pressure, and limb movements [1, 2]. Lacking objective data support, these methods also face challenges in continuous monitoring due to low specificity and sensitivity [1]. Such limitations may lead to inaccurate and untimely assessments, potentially resulting in either excessive or insufficient anesthesia, which significantly impacts patients' mental health, disease recovery, and long-term survival rates [1].

The Bispectral Index (BIS) offers an objective and precise method for monitoring the depth of anesthesia [3], which is a crucial component of some Enhanced Recovery After Surgery (ERAS) guidelines [4, 5]. ERAS is an evidence-based approach to surgical care aimed at improving the quality of perioperative care and supporting quick recovery [5, 6]. By quantifying the excitatory or inhibitory states of the cerebral cortex through analyzing power and frequency in an electroencephalogram (EEG), BIS provides a numerical value that corresponds to a specific level of consciousness, reflecting the functional status of the cerebral cortex [3]. This enables the continuous, noninvasive monitoring of anesthesia depth throughout the perioperative period, aligning with ERAS goals to optimize patient recovery, minimize complications, and enhance recovery speed.

There is substantial evidence indicating that the use of BIS monitoring during anesthesia can decrease the occurrence of adverse clinical events, supporting the ERAS objective of improving patient outcomes and expediting recovery. However, some findings revealed conflicting results regarding the use of BIS monitoring [7]. This systematic review and meta-analysis aimed to comprehensively evaluate the effectiveness of BIS monitoring for depth of anesthesia compared to traditional clinical parameters.

## 2. Materials and Methods

### 2.1. Search Strategies

From the database inception to April 20, 2023, the researchers systematically searched scientific information sources in Ovid-MEDLINE, Cochrane, and PubMed. The search strategy included keywords such as (BIS monitoring/BIS) AND (Anesthesia, General OR Anesthetics) AND (Postoperative delirium OR Anesthesia dosage OR Neurological function OR Postoperative nausea and vomiting OR Abnormal blood pressure OR Anesthesia recovery period (eye opening; orientation force recovery time; extubation time; time for hospital discharge) OR Delayed Emergence from Anesthesia OR Mortality OR Operative Time Surgery time OR Postoperative Cognitive Complication OR Intraoperative Awareness) AND (Randomized Controlled Trial). In addition, we conducted searches of clinical trial registers and grey literature and manually reviewed reference lists of included studies as well as related review articles.

### 2.2. Selection Criteria

#### 2.2.1. Inclusion Criteria

The inclusion criteria were restricted to randomized controlled trials (RCTs) in English without restrictions on gender or age. The control groups employed conventional methods for monitoring anesthetic depth, while the intervention groups utilized BIS monitoring during anesthesia. The outcome indicators are outlined in Table 1.

#### 2.2.2. Exclusion Criteria

The exclusion criteria covered duplicate publications, reviews, or commentary-type studies; animal experiments; studies with unclear outcome observation indicators; and studies with incomplete or inaccessible data.

### 2.3. Data Extraction

Two researchers independently and blindly screened and extracted the data, including the first author of the study, year of publication, sample sizes of the intervention and control groups, type of surgery, and outcomes. When studies with indeterminate information were encountered, an independent adjudication was performed by a third researcher.

### 2.4. Quality Assessment

Cochrane risk-of-bias tool was utilized to evaluate the quality of the included literature across seven indicators: random sequence generation, allocation concealment, blinding of participants and personnel, blinding of outcome assessment, incomplete outcome data, selective reporting, and other bias.

### 2.5. Statistical Analysis

Continuous data were represented as mean differences (MDs) and standardized mean differences (SMDs) with 95% confidence intervals (CIs), while count data were expressed as relative risks (RRs) with a 95% CI.

Heterogeneity was comprehensively assessed using the *I*^2^ statistic and *Q*-test. *I*^2^ values greater than 50% or a *Q*-test score with a *P* value less than 0.05 indicated high heterogeneity. The random effects model was employed for effect size merging in cases with high heterogeneity, while the fixed effects model was used for other cases.

The meta-analysis results were visually presented through forest plots. Funnel plots and Egger's test were employed to assess publication bias. Sensitivity analysis and subgroup analysis were conducted for further exploration in studies with high heterogeneity. All results with a *P* value less than 0.05 were considered statistically significant. The R 4.0 software was utilized for the data analysis.

## 3. Results

### 3.1. Study Selection and Study Characteristics

**Figure 1:**
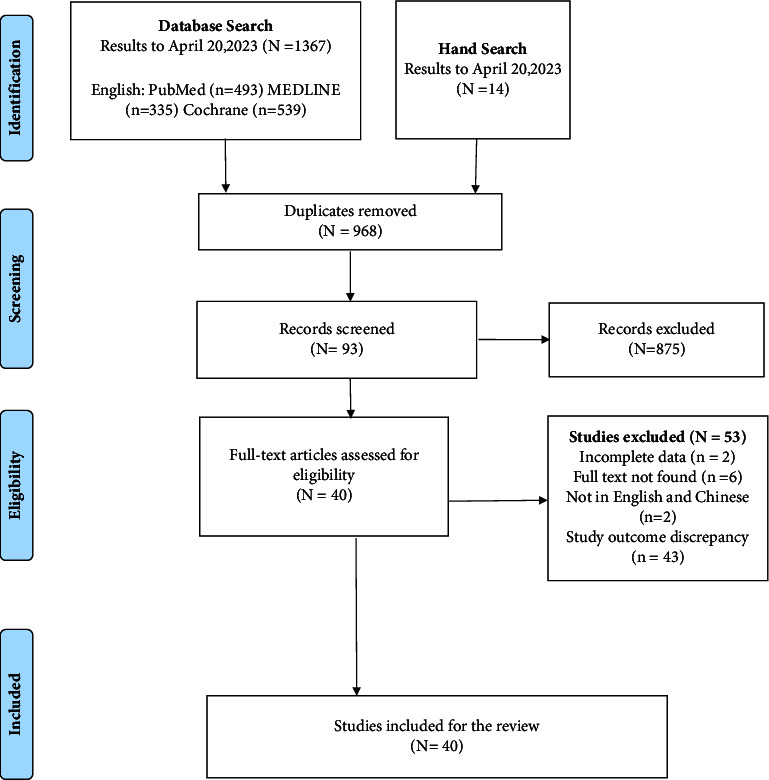
Study flow diagram.

A comprehensive search of databases yielded a total of 1367 articles, distributed across PubMed (493), MEDLINE (335), and Coch rane (539), supplemented by an additional 14 relevant articles from other sources. After removing duplicates, 968 articles remained. Subsequent scrutiny of the titles and abstracts led to the exclusion of 875 articles that were unrelated to the research topic. A detailed review of the full texts resulted in the exclusion of articles that did not meet the inclusion criteria, ultimately culminating in the inclusion of 40 studies (Figure 1). The characteristics of the included studies are presented in Table 2.

### 3.2. Risk of Bias

**Figure 2:**
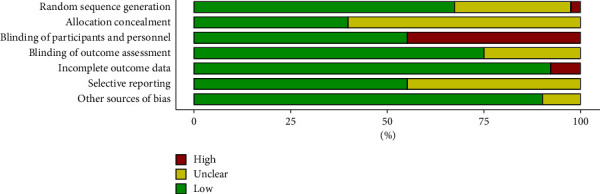
Methodological quality assessment for all included studies.

**Figure 3:**
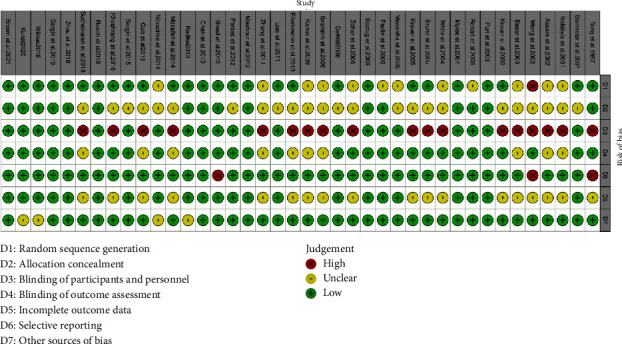
Methodological quality items for each included study.

The quality of the included articles was assessed using the Cochrane risk-of-bias tool, which involved the evaluation of seven indicators for each source from the literature (Figure 2). Among selected studies, 13 studies did not clearly report whether a randomization method was employed, 24 studies did not clearly report whether allocation concealment was implemented, 10 studies did not report whether the outcome assessors were blinded, and most of the studies did not report whether the participants and personnel were blinded (Figure 3).

### 3.3. Meta-Analysis Results

The meta-analysis results of the included studies about perioperative complications, anesthesia recovery period, and anesthetic dosage are summarized in Table 3.

#### 3.3.1. Perioperative Complications

*(1) Postoperative Delirium*. The intervention group comprised 1580 individuals, while the control group included 1586 individuals. Utilizing BIS monitoring during anesthesia did not significantly reduce postoperative delirium compared to when conventional clinical monitoring was used (RR = 0.82, 95% CI: 0.63∼1.08, *P* = 0.16, and *I*^2^ = 66.7%) (Supplementary 2.1 (S2.1) Figure S1).

*(2) Postoperative Nausea and Vomiting*. The intervention group included 1556 individuals, and the control group included 1645 individuals. The use of BIS monitoring during anesthesia did not significantly decrease the occurrence of postoperative nausea and vomiting compared to when conventional clinical monitoring was used (RR = 1.07, 95% CI: 0.89∼1.28, *P*=0.49, and *I*^2^ = 18%) (S2.1Figure S2).

*(3) Abnormal Blood Pressure*. The intervention group comprised 1723 individuals, while the control group included 1750 individuals. Using BIS monitoring during anesthesia did not result in a significant difference in the incidence of abnormal blood pressure, compared to the results observed with conventional clinical monitoring (RR = 1.03, 95% CI: 0.97∼1.10, *P* = 0.33, and *I*^2^ = 20.6%) (S2.1 and Figure S3).

*(4) Intraoperative Awareness*. The intervention group included 4623 individuals, while the control group comprised 4036 individuals. The statistical results did not indicate a significant difference in intraoperative awareness between the use of BIS monitoring and conventional monitoring during anesthesia (RR = 0.63, 95% CI: 0.26∼1.53, *P*=0.30, and *I*^2^ = 56%) (S2.1 and Figure S4).

*(5) POCD*. The intervention group included 2055 individuals, while the control group included 2090 individuals. The use of BIS monitoring during anesthesia resulted in a significant reduction in the risk of POCD compared to when conventional monitoring was used (RR = 0.85, 95% CI: 0.73∼0.99, *P* = 0.04, and *I*^2^ = 22.8%) (S2.1 and Figure S5).

*(6) Mortality*. The intervention group included 2525 individuals, and the control group comprised 2542 individuals. The statistical results did not reveal a significant difference in mortality between the use of BIS monitoring and conventional monitoring during anesthesia (RR = 0.66, 95% CI: 0.29∼1.50, *P* = 0.32, and *I*^2^ = 62.9%) (S2.1 and Figure S6).

#### 3.3.2. Anesthesia Recovery Period

*(1) Eye-Opening Time*. The intervention group included 1940 individuals, while the control group included 1966 individuals. The statistical results indicate that the use of BIS monitoring significantly shortens the patients' eye-opening times compared to when conventional anesthesia monitoring is used (MD = −1.34, 95% CI: −2.06∼−0.61, *P* < 0.01, and *I*^2^ = 76%) (S2.1 and Figure S7).

*(2) Orientation Force Recovery Time*. Regarding the analysis of the orientation force recovery time, the intervention group included 135 individuals and the control group included 134 individuals. In comparison to conventional anesthesia monitoring, the utilization of BIS monitoring can significantly reduce patients' orientation force recovery times (MD = −1.99, 95% CI: −3.62∼−0.36, *P*=0.02, and *I*^2^ = 88%) (S2.1 and Figure S8).

*(3) Extubation Time*. The intervention group included 450 individuals, while the control group included 450 individuals. Using BIS monitoring significantly shortens the extubation time for patients compared to when conventional anesthesia monitoring is used (MD = −2.54, 95% CI: −3.50∼−1.58, *P* < 0.01, and *I*^2^ = 74.6%) (S2.1 and Figure S9).

*(4) PACU Stay Duration*. The intervention group included 8500 individuals, and the control group included 5889 individuals. The implementation of BIS monitoring significantly reduces the PACU stay time for surgical patients compared to when conventional anesthesia monitoring is used (MD = −7.11, 95% CI: −12.67∼−1.55, *P* = 0.01, and *I*^2^ = 90.1%) (S2.1 and Figure S10).

*(5) Surgery Time*. The intervention group comprised 1536 individuals, while the control group comprised 1603 individuals. Utilizing BIS monitoring in anesthesia did not show a significant difference in the surgery time compared to when conventional monitoring methods were used (MD = 0.11, 95% CI: −1.65∼1.87, *P* = 0.90, and *I*^2^ = 78.9%) (S2.1 and Figure S11).

#### 3.3.3. Anesthetic Dosage

In the anesthetic dosage meta-analysis, the intervention group included 23878 individuals while the control group comprised 16160 individuals. Compared to conventional monitoring during anesthesia, the use of BIS monitoring resulted in a significant reduction in the anesthetic dosage (SMD = −0.39, 95% CI: −0.63∼−0.15, *P* < 0.01, and *I*^2^ = 98.4%) (S2.1 and Figure S12).

A subgroup analysis was performed for commonly used anesthetics during surgery, including propofol, fentanyl, and other types of drugs (S2.1 and Figure S13). Among all these anesthetic drugs, the study results indicated that the use of BIS monitoring did not significantly reduce the propofol and fentanyl dosages during anesthesia.

### 3.4. Publication Bias

The meta-analysis of eye-opening time, extubation time, and PACU stay duration exhibited publication bias (Table 4). The funnel plot of outcomes is shown in Supplementary 2.2.

## 4. Discussion

Our study systematically assessed the study comparing the use of BIS monitoring to traditional methods of measuring anesthesia depth. This study comprehensively analyzed the clinical effectiveness of using BIS monitoring during anesthesia, including its impact on perioperative complications, anesthesia recovery period, and anesthetic dosage. The results showed that using BIS to monitor the depth of anesthesia for patients undergoing general anesthesia significantly reduced the risk of POCD, shortened the eye-opening time, orientation force recovery time, extubation time, and PACU stay duration and lowered the anesthesia drug dosage.

Our study found a significant reduction in the risk of POCD when BIS monitoring was used during anesthesia, which was similar to the prior studies [7, 34]. An RCT found that in elderly patients undergoing major noncardiac surgeries, the use of BIS monitoring reduced the risk of POCD by 31% three months after surgery [34]. Another meta-analysis suggested that anesthesia depth control using BIS had a significant 3% reduction in the risk of POCD [7]. The potential mechanism could be that BIS monitoring during anesthesia leads to a reduction in cerebral metabolism and the stress response to surgery, which in turn may decrease the POCD [34].

It is noteworthy that our study did not find a reduction in the risk of postoperative delirium, which is highly associated with POCD when using BIS during anesthesia. This finding contrasted with the results of some previous studies. For instance, a meta-analysis conducted by Shan et al., which included 8 studies, indicated a significant reduction in postoperative delirium when BIS monitoring was utilized during anesthesia [48]. The discrepancy between our study and prior studies may be attributed to variations in study participants, types of surgery, and depths of anesthesia achieved using BIS monitoring. For instance, a systematic review focused on the prevention and treatment of delirium in adult patients undergoing cardiac surgery established that the effects of dexmedetomidine on delirium are consistent with the findings associated with BIS monitoring [49]. It suggests that further studies need to specifically evaluate the effect of BIS monitoring on anesthetic depth. In addition, individuals who are older, male, and have conditions such as dementia are more likely to experience postoperative delirium [50]. Future studies should specifically evaluate delirium in these high-risk groups.

In terms of the meta-analysis results on the anesthesia recovery period, our study found that using BIS monitoring could significantly shorten the eye-opening time, orientation force recovery time, extubation time, and PACU stay duration, which aligned with the results of prior studies [7, 51]. For example, Oliveira et al.'s meta-analysis of using BIS during anesthesia, encompassing 17 studies published up to 2015, showed that the use of BIS monitoring during anesthesia significantly reduced the extubation time, orientation force recovery time, and the time taken to leave the operating room [7]. Another meta-analysis demonstrated that BIS-guided anesthesia shortened early recovery times regardless of the anesthetic drugs used [51]. This may be due to the fact that using BIS to reduce anesthetic dosage to optimal levels at the end of surgery accelerates anesthesia recovery time.

In the context of anesthesia drug dosage, our study showed that anesthetic dosage was significantly reduced when using BIS, consistent with the results of previous studies. However, our subgroup analysis showed that the use of BIS monitoring did not significantly reduce the dosage of propofol and fentanyl during anesthesia. Besides, some prior studies found that using different anesthetics under BIS monitoring has varied impacts on the occurrence of postoperative adverse events. For example, the meta-analysis conducted by Lewis et al. revealed that the use of propofol, desflurane, isoflurane, and sevoflurane during anesthesia had diverse effects on postoperative delirium, the postoperative eye-opening time, orientation force recovery time, and PACU stay time [51]. Furthermore, using BIS with different anesthesia methods, such as intravenous and inhalation anesthetics, may lead to a different effect. Further studies are needed to more thoroughly explore the effectiveness of BIS under different anesthetic drugs and methods.

Our study comprehensively evaluated the effectiveness of using BIS during anesthesia and provided up-to-date evidence. In addition, our findings provide robust support for integrating BIS into ERAS protocols, which emphasize minimizing the impact of anesthetic agents and techniques on organ function [5]. Our results underscore the value of BIS in aligning with these ERAS goals by offering precise control over anesthesia depth.

There are also some limitations in our study. First, the included studies encompassed a wide variety of surgical types, which could potentially limit the precision of our meta-analysis, particularly in assessing outcomes like mortality risk that are significantly influenced by the type of surgery. Second, the study participants and clinical settings included in our study were excessively broad, which may lead to a lack of specificity. As a result, it may be challenging to broadly generalize these results across all clinical anesthesia settings. Third, this study only analyzed the use of BIS monitoring during anesthesia, whereas many studies have described the occurrence of clinical adverse events based on different BIS monitoring values, potentially impacting the study's results. There is a need to further refine BIS values into distinct subgroups for more detailed analysis. Fourth, many included studies did not report allocation concealment and various biases, including selection bias and implementation bias, may have affected the authenticity and objectivity of the conclusions. Particularly, due to the difficulty of implementing blinding in the use of BIS-monitoring devices, this study may have underestimated the effects of lack of blinding.

## 5. Conclusion

The results showed that the use of BIS monitoring during anesthesia has a significant impact on clinical effectiveness, particularly in reducing POCD, shortening eye-opening time, orientation force recovery time, extubation time, PACU stay duration, and decreasing anesthesia drug dosage. Our study provided updated evidence for using BIS during anesthesia. However, it may be challenging to broadly generalize these results across all clinical anesthesia settings because our study was excessively broad. Further research is needed to be more specific in discussing the effectiveness of using BIS to enhance the certainty of evidence.

## Figures and Tables

**Table 1:** Considered outcomes in the study.

Domains	Outcomes
Perioperative complications	Postoperative delirium
	Postoperative nausea and vomiting
	Abnormal blood pressure
	Intraoperative awareness
	Postoperative cognitive dysfunction (POCD)
	Mortality

Anesthesia recovery period	Eye-opening time
	Orientation force recovery time
	Extubation time
	Postanesthesia care unit (PACU) stay duration
	Surgery time

Anesthetic dosage	Anesthetic dosage

**Table 2:** The characteristics of the included studies.

First author (year)	Country	Age	Type of surgery	*N* (intervention/control)	Outcomes
Gan et al. (1997) [8]	America	37–43	General anesthesia surgery	240 (115/125)	Abnormal blood pressure/eye-opening time/extubation time
Bannister et al. (2001) [9]	America	0–18	Inguinal hernia repair (0–3 y)/tonsillectomy/adenoidectomy (3–18 yr)	202 (97/105)	Extubation time/PACU stay duration/surgery time
Nelskyla et al. (2001) [10]	Finland	18–50	Gynecological laparoscopic surgery	62 (32/30)	Postoperative nausea and vomiting/anesthesia dosage/orientation force recovery time/extubation time/surgery time
Assare et al. (2002) [11]	Sweden	18–65	Elective arthroscopic surgery	40 (20/20)	Postoperative nausea and vomiting/intraoperative awareness/surgery time
Wong et al. (2002) [12]	Canada	64–76	Orthopedic knee or hip replacement surgery	60 (29/31)	Intraoperative awareness/anesthesia dosage/eye-opening time/orientation force recovery time/PACU stay duration/surgery time
Basar et al. (2003) [13]	Turkey	41	Open abdominal surgery	60 (30/30)	Eye-opening time
Kreuer et al. (2003) [14]	Germany	18–80	Minor orthopedic surgery	80 (40/40)	Intraoperative awareness/anesthesia dosage/eye-opening time
Puri and Murthy (2003) [15]	India	18–70	Arterial transplantation (CABG) or cardiopulmonary bypass replacement (CPB) or valve replacement	30 (14/16)	Abnormal blood pressure/intraoperative awareness/eye opening/extubation time/surgery time
Recart et al. (2003) [16]	America	31–64	Endoscopic general surgery	60 (30/30)	Anesthesia dosage/eye-opening time/extubation time/PACU stay duration/surgery time
Myles et al. (2004) [17]	Australia	>18	Routine operation	2463 (1225/1238)	Abnormal blood pressure/intraoperative awareness/mortality/anesthesia dosage/eye opening/PACU stay duration
White et al. (2004) [18]	America	51	Gynecological laparoscopic surgery	40 (20/20)	Postoperative nausea and vomiting/intraoperative awareness/anesthesia dosage/eye-opening time/orientation force recovery time/extubation time/PACU stay duration/surgery time
Bruhn et al. (2005) [19]	Germany	18–80	Minor surgery	142 (71/71)	Postoperative nausea and vomiting/intraoperative awareness/eye opening/PACU stay duration/surgery time
Kreuer et al. (2005) [20]	Germany	18–80	Minor orthopedic surgery	80 (40/40)	Eye-opening time/extubation time
Messieha et al. (2005) [21]	America	2–18	Dental rehabilitation	29 (15/14)	Extubation time/PACU stay duration/surgery time
Pavlin et al. (2005) [22]	America	46.5	General anesthesia surgery	1580 (749/831)	Postoperative nausea and vomiting/anesthesia dosage/PACU stay duration/surgery time
Boztug et al. (2006) [23]	Turkey	18–75	Surgical operation	47 (24/23)	Anesthesia dosage/eye-opening time/extubation time/PACU stay duration/surgery time
Zohar et al. (2006) [24]	Israel	65–83	Elective transurethral surgery	50 (25/25)	Intraoperative awareness/anesthesia dosage/orientation force recovery time/PACU stay duration/surgery time/delayed emergence from anesthesia
DeWitt (2008) [25]	America	37–63	Whole crowd	44 (24/20)	Anesthesia dosage
Ibraheim et al. (2008) [26]	Saudi Arabia	34.5–46.5	Laparoscopic gastric banding	30 (15/15)	Eye-opening time/extubation time/delayed emergence from anesthesia
Mostafa et al. (2009) [27]	Egypt	45–60	Abdominal surgery	60 (30/30)	Intraoperative awareness/eye-opening time/orientation force recovery time
Ellerkmann et al. (2010) [28]	Germany	18–80	Upper or lower limb regional anesthesia surgery	60 (30/30)	Intraoperative awareness/eye-opening time
Liao et al. (2011) [29]	America	3–12	Urological surgery	106 (52/54)	Postoperative nausea and vomiting/anesthesia dosage/eye-opening time/surgery time
Zhang et al. (2011) [30]	China	>18	General anesthesia surgery	5228 (2919/2309)	Intraoperative awareness
Mashour et al. (2012) [31]	America	41–64	General anesthesia surgery	9460 (6076/3384)	Anesthesia dosage/PACU stay duration
Persec et al. (2012) [32]	America	25–84	Major abdominal surgery	45 (20/20)	Anesthesia dosage/extubation time/surgery time
Bresil et al. (2013) [33]	Denmark	1–65	Selective ear, nose, and throat surgery	157 (79/78)	Eye opening time/surgery time
Chan et al. (2013) [34]	China	>60	Selective major surgery	921 (462/459)	Postoperative delirium/POCD/anesthesia dosage
Radtke (2013) [35]	Germany	>60	Surgical procedures	1155 (575/580)	Postoperative delirium/POCD/mortality
Mozafari et al. (2014) [36]	Iran	18–65	Elective abdominal surgery	333 (163/170)	Intraoperative awareness
Nitzschke et al. (2014) [37]	Germany	65.7	Elective pump heart surgery	67 (31/29)	Surgery time
Guo et al. (2015) [38]	China	18–65	Selective escharotomy	40 (20/20)	Intraoperative awareness/eye-opening time
Sargin et al. (2015) [39]	New Zealand	6∼16	Dental treatment under general anesthesia/moderate developmental delay	40 (20/20)	Eye-opening time/extubation time/PACU stay duration/surgery time
Khoshrang et al. (2016) [40]	Iran	15–65	Open kidney surgery	96 (48/48)	Surgery time
Rusch et al. (2018) [41]	Germany	48	Minor elective surgery	235 (120/115)	Abnormal blood pressure
Makkar et al. (2018) [42]	India	20–60	Lumbar surgery	44 (22/22)	Intraoperative awareness
Zhou et al. (2018) [43]	China	65–75	Colon cancer patient	81 (41/40)	Postoperative delirium/anesthesia dosage/surgery time
Sargin et al. (2019) [44]	Turkey	18–70	Colonoscopy	100 (50/50)	Anesthesia dosage
Wildes et al. (2019) [45]	America	>60	Major operation	1232 (614/618)	Postoperative delirium/postoperative nausea and vomiting/mortality
Kunst et al. (2020) [46]	Britain	>65	Extracorporeal circulation elective coronary artery bypass grafting	82 (42/40)	Postoperative delirium
Brown et al. (2021) [47]	America	≥65	Lumbar surgery	217 (111/106)	POCD/mortality/PACU stay duration/surgery time

**Table 3:** The results of the included studies in the meta-analysis.

Domains	Outcomes	*N*	Heterogeneity		Effect model	Meta-analysis results		*P*
			*I* ^2^ (%)	*P*		Effect value	Combined effect size (95% CI)	
Perioperative complications	Postoperative delirium	6	66.7	0.01	Random	RR	0.82 (0.63, 1.08)	0.16
	Postoperative nausea and vomiting	7	18.4	0.29	Fixed	RR	1.07 (0.89, 1.28)	0.49
	Abnormal blood pressure	7	20.6	0.27	Fixed	RR	1.03 (0.97, 1.10)	0.33
	Intraoperative awareness	14	55.6	0.08	Random	RR	0.63 (0.26, 1.53)	0.30
	POCD	5	22.8	0.27	Fixed	RR	0.85 (0.73, 0.99)	0.04
	Mortality	4	62.9	0.04	Random	RR	0.66 (0.29, 1.50)	0.32

Anesthesia recovery period	Eye-opening time	22	76.1	<0.01	Random	MD	−1.34 (−2.06, −0.61)	<0.01
	Orientation force recovery time	5	87.8	<0.01	Random	MD	−1.99 (−3.62, −0.36)	0.02
	Extubation time	14	74.6	<0.01	Random	MD	−2.54 (−3.50, −1.58)	<0.01
	PACU stay duration	15	90.1	<0.01	Random	MD	−7.11 (−12.67, −1.55)	0.01
	Surgery time	25	78.9	<0.01	Random	MD	0.11 (−1.65, 1.87)	0.90

Anesthetic dosage	Anesthetic dosage	27	98.4	.	Random	SMD	−0.39 (−0.63, −0.15)	<0.01

*Notes*. Relative risk ratio, RR; mean difference, MD; standard mean difference, SMD; “.”, cannot be calculated.

**Table 4:** The results of publication bias.

Domains	Outcomes	Egger test	Publication bias
Perioperative complications	Postoperative delirium	0.56	No
	Postoperative nausea and vomiting	0.90	No
	Abnormal blood pressure	0.22	No
	Intraoperative awareness	0.63	No
	POCD	0.81	No
	Mortality	0.27	No

Anesthesia recovery period	Eye-opening time	0.05	Yes
	Orientation force recovery time	0.47	No
	Extubation time	<0.01	Yes
	PACU stay duration	0.01	Yes
	Surgery time	0.37	No

Anesthesia dosage	Anesthetic dosage	0.07	No

## Data Availability

All data generated or analyzed during this study are included within the article.

## Supplementary Materials

Supplementary Materials Supplementary 1: PRISMA 2020 Checklist; Supplementary 2: Supplementary 2.1: Forest Plots and Supplementary 2.2: Funnel Plots. Figure S1: meta-analysis results of postoperative delirium. Figure S2: meta-analysis results of postoperative nausea and vomiting. Figure S3: meta-analysis results of abnormal blood pressure. Figure S4: meta-analysis results of intraoperative awareness. Figure S5: meta-analysis results of postoperative cognitive dysfunction. Figure S6: meta-analysis results of mortality. Figure S7: meta-analysis results of eye-opening time. Figure S8: meta-analysis results of orientation force recovery time. Figure S9: meta-analysis results of extubation time. Figure S10: meta-analysis results of PACU stay duration. Figure S11: meta-analysis results of surgery time. Figure S12: meta-analysis results of anesthetic dosage. Figure S13: subgroup meta-analysis results of anesthetic dosage.
