# Supplementary material for: Effectiveness Assessment of Bispectral Index Monitoring Compared with Conventional Monitoring in General Anesthesia: A Systematic Review and Meta-Analysis
Source: Anesthesiol Res Pract. 2024 Aug 7;2024:5555481. doi: 10.1155/2024/5555481 (PMC11325011; doi:10.1155/2024/5555481)

Supplementary 2: Forest Plots and Funnel Plots

Supplementary 2.1 Forest Plot
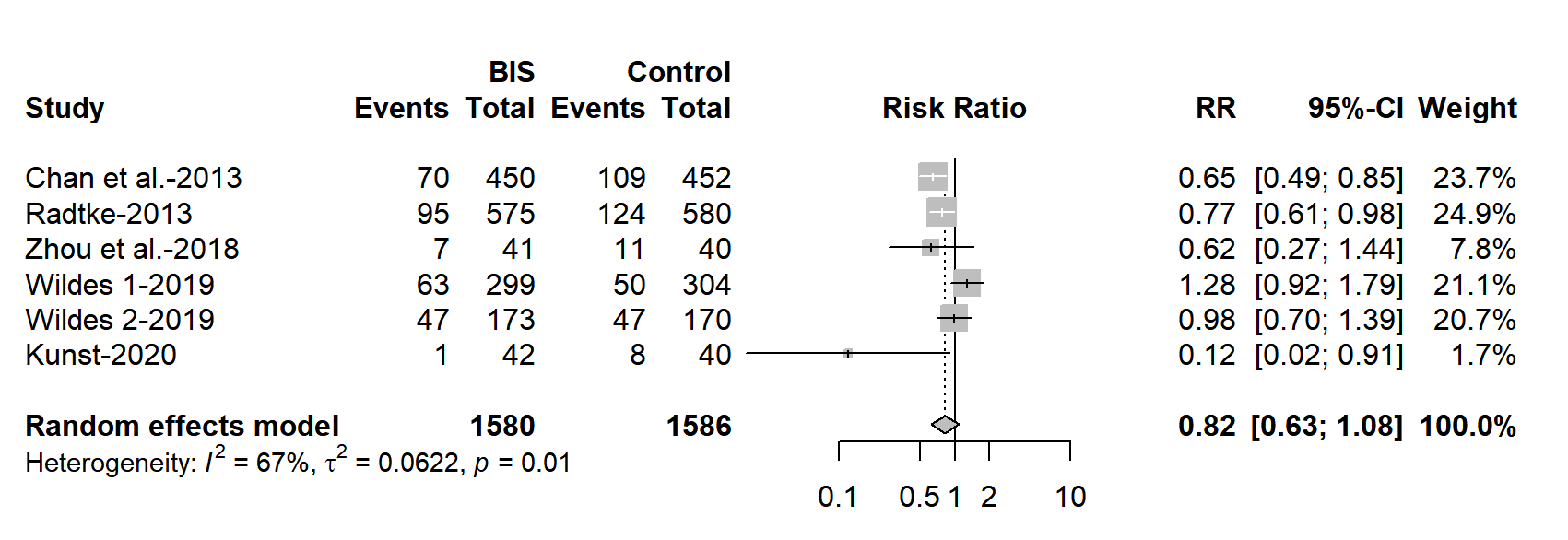
s


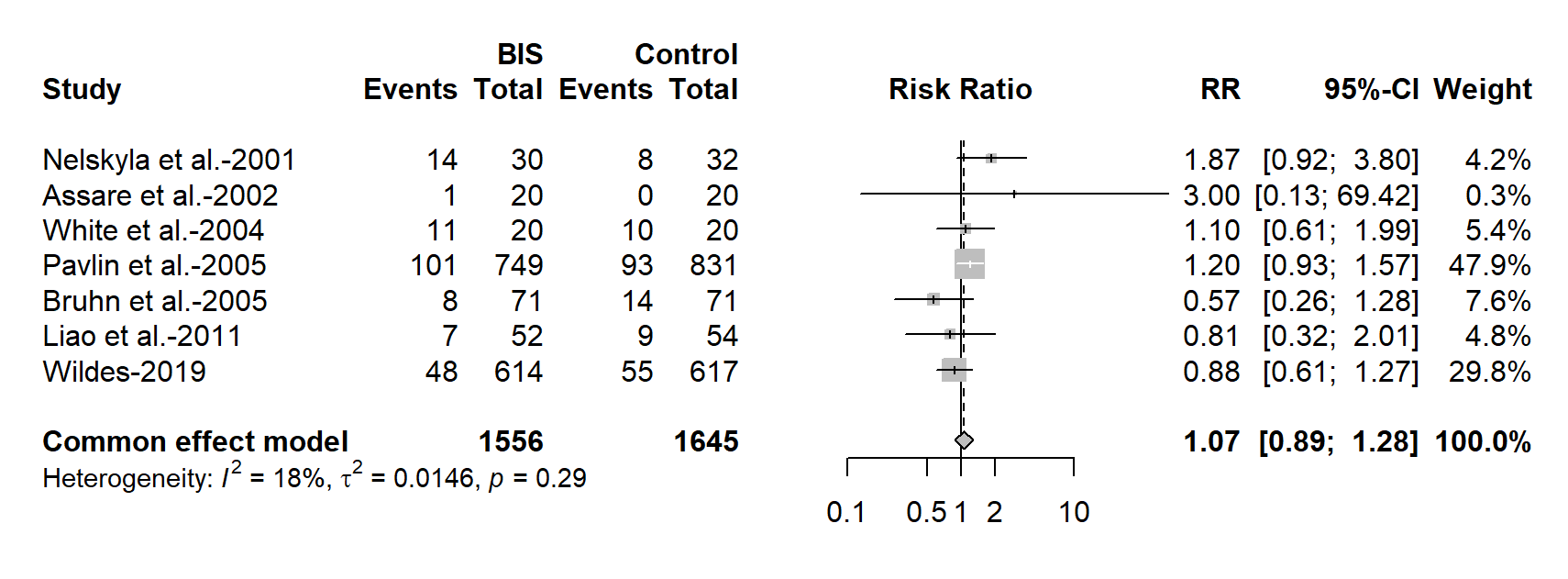
**Figure S1**. Meta-analysis results of postoperative delirium

**Figure S2**. Meta-analysis results of postoperative nausea and vomiting


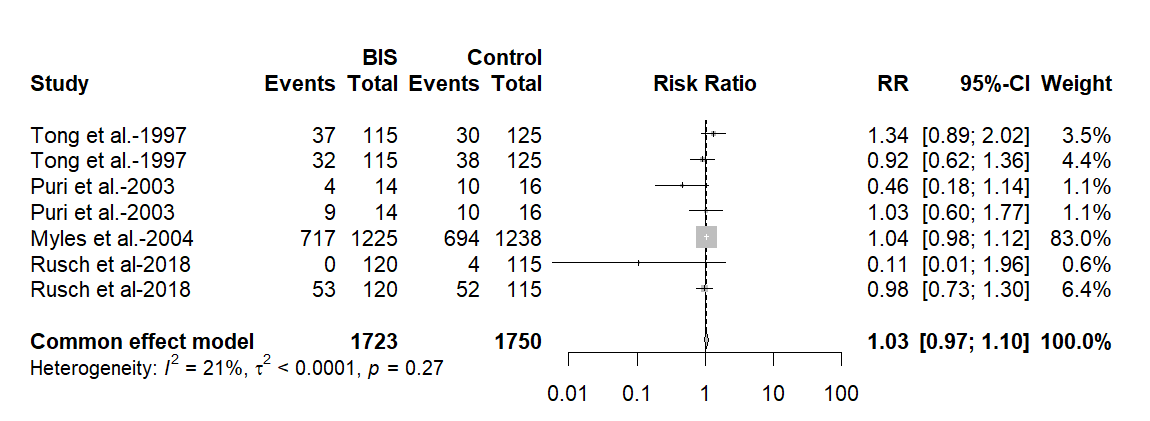


**Figure S3**. Meta-analysis results of abnormal blood pressure


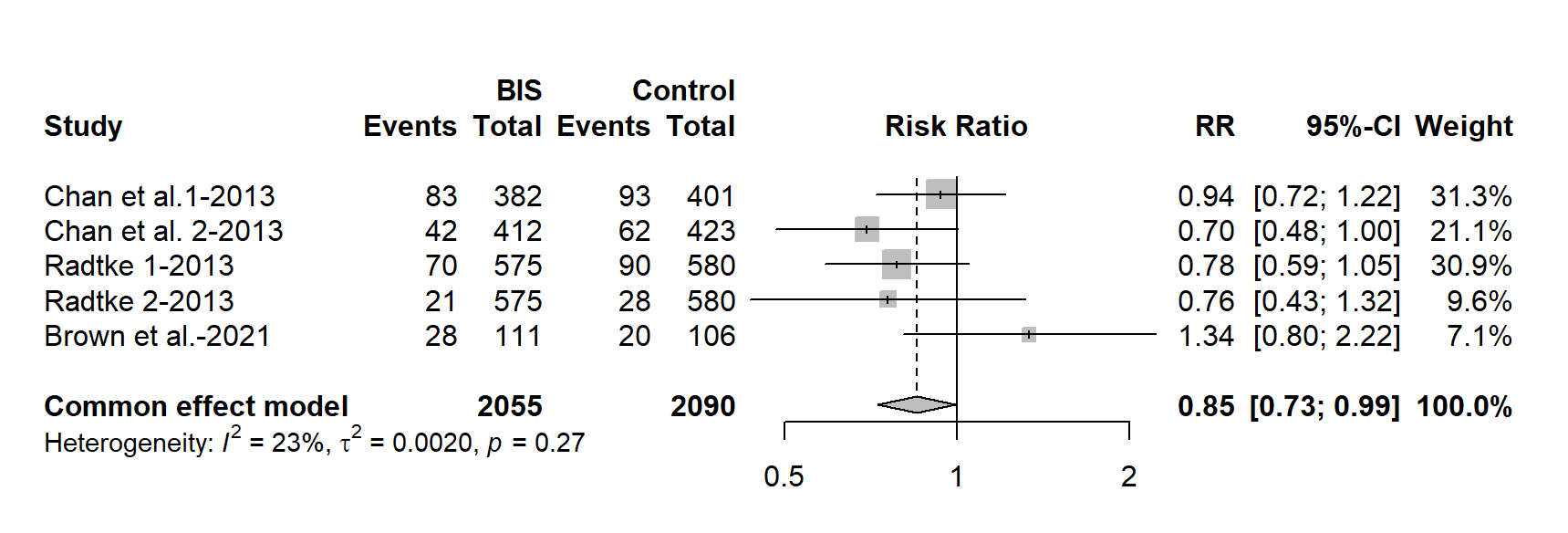
**Figure S4**. Meta-analysis results of intraoperative awareness
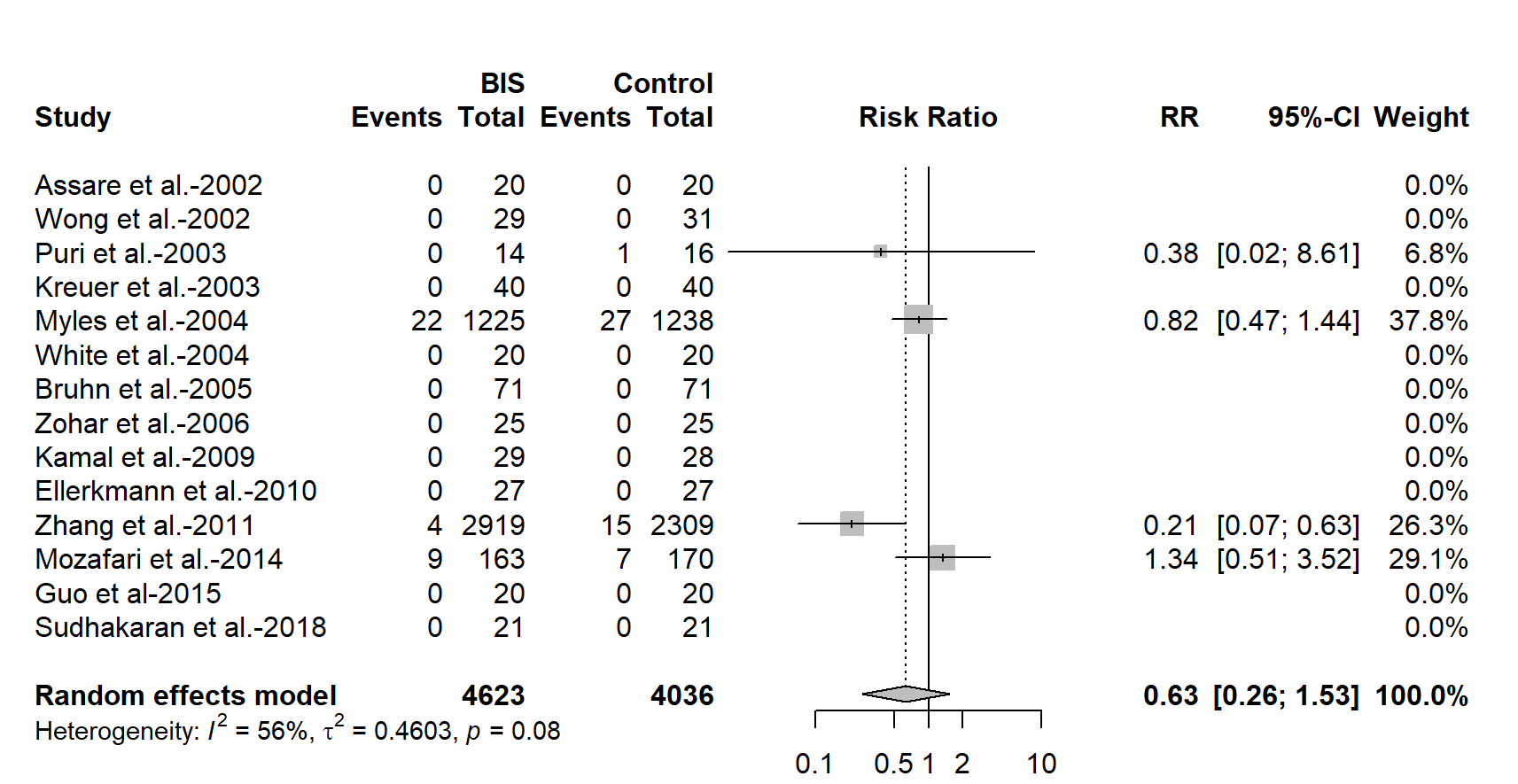


**Figure S5.** Meta-analysis results of postoperative cognitive dysfunction


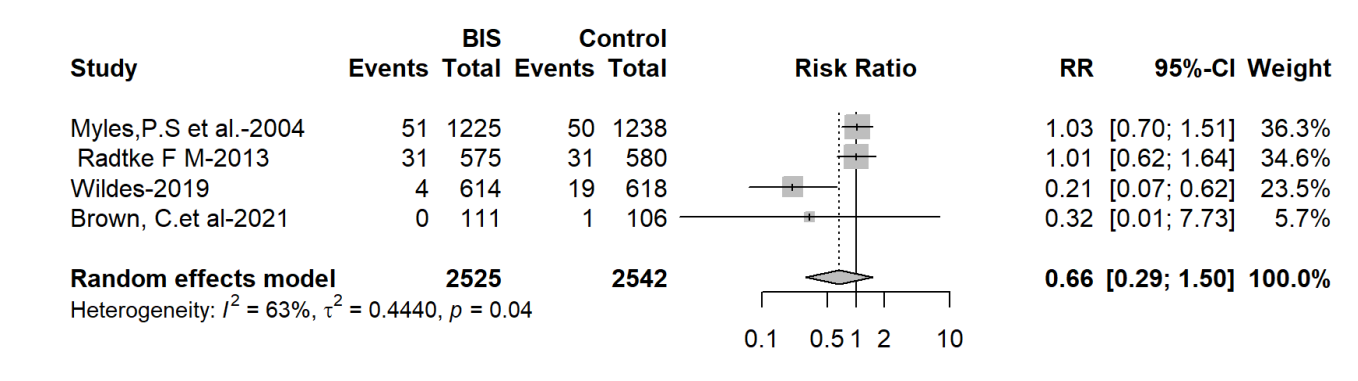


**Figure S6.** Meta-analysis results of mortality


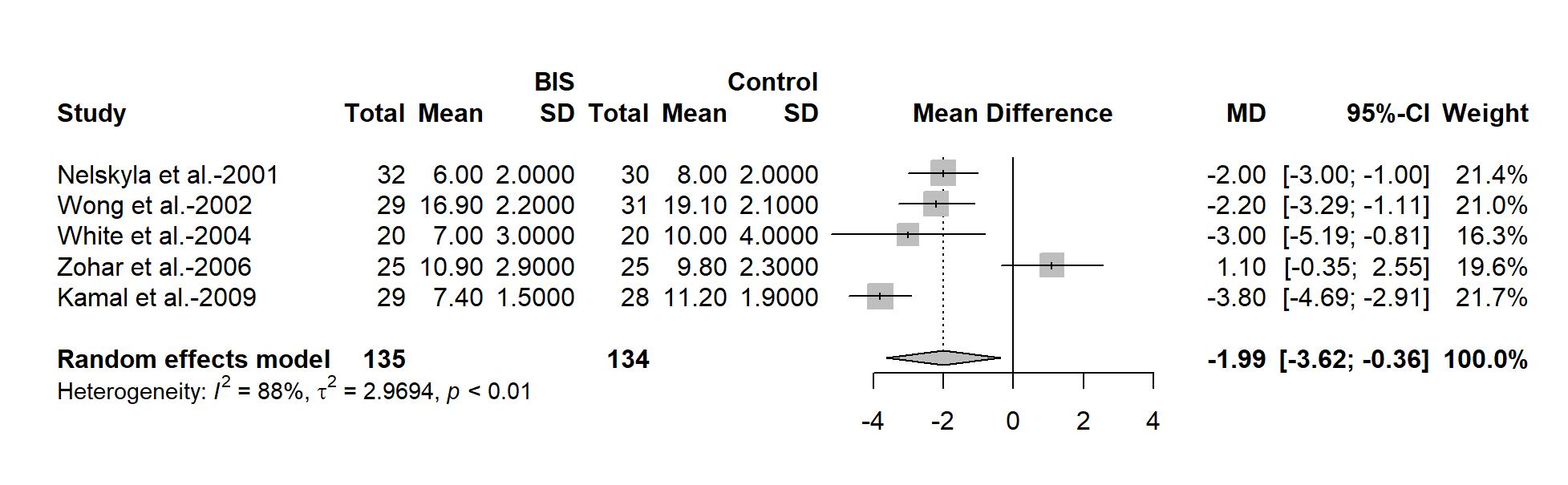

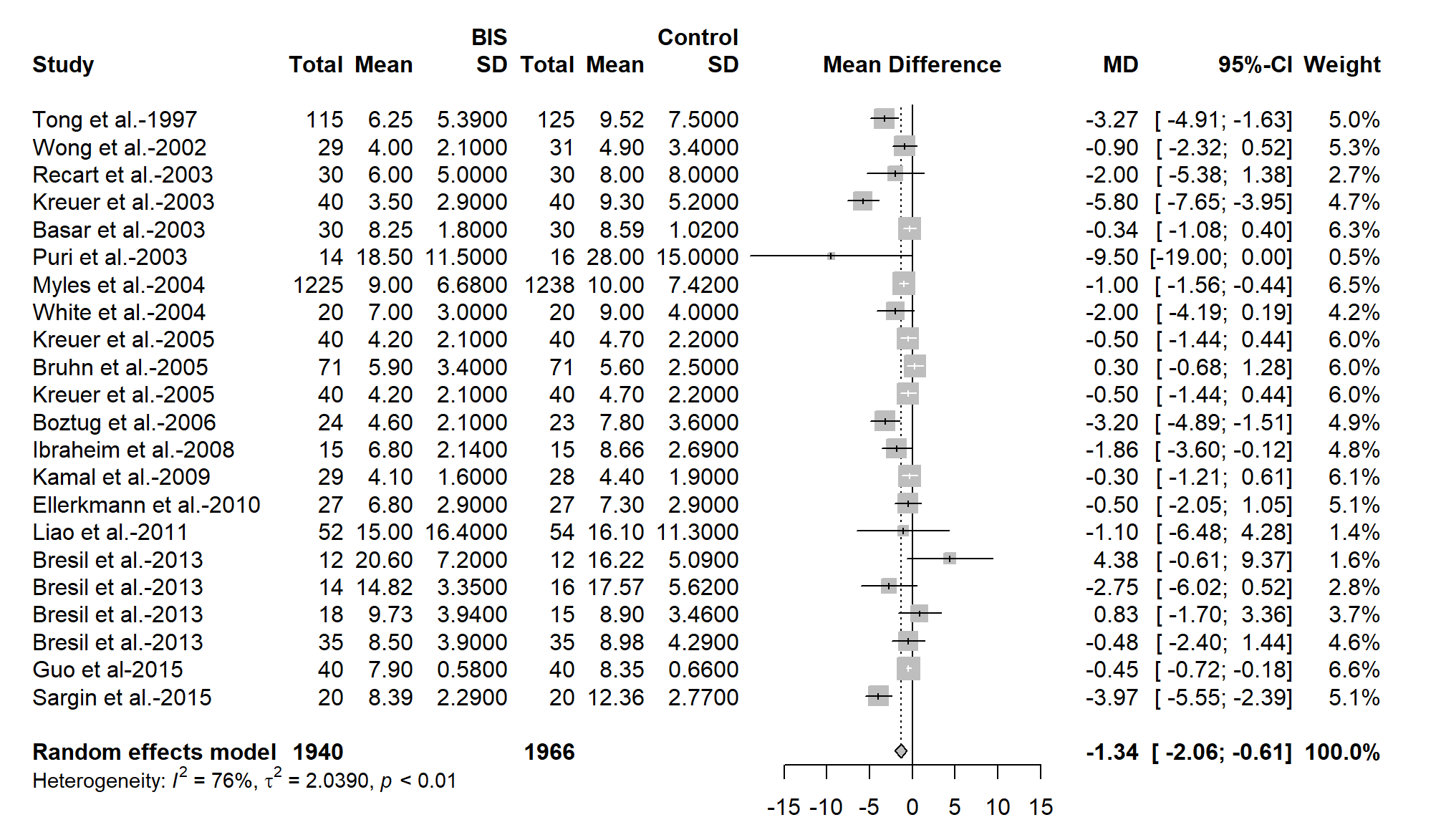
**Figure S7.** Meta-analysis results of eye opening time

**Figure S8.** Meta-analysis results of orientation force recovery time


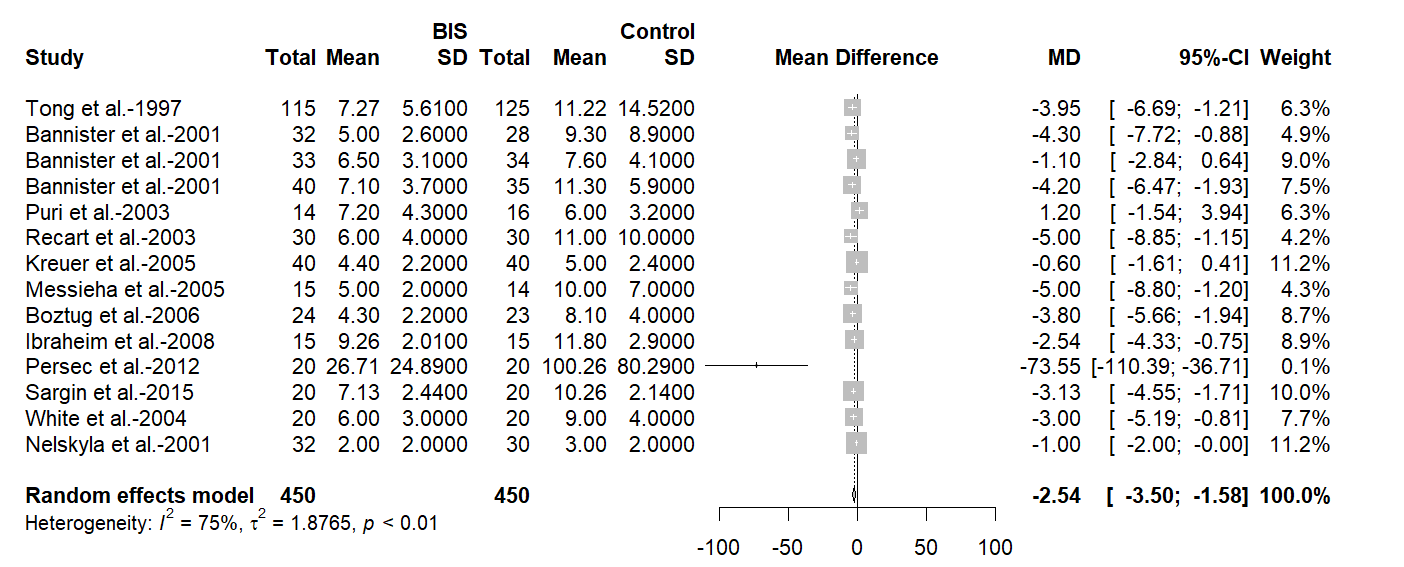


**Figure S9.** Meta-analysis results of extubation time


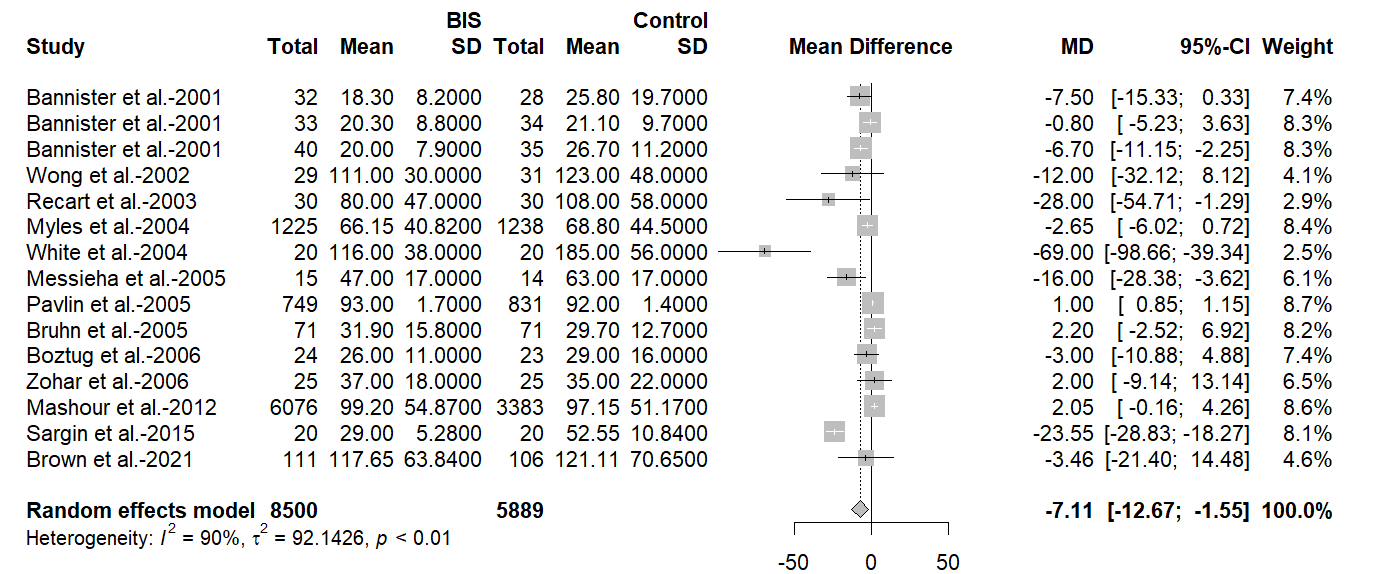


**Figure S10.** Meta-analysis results of PACU stay duration


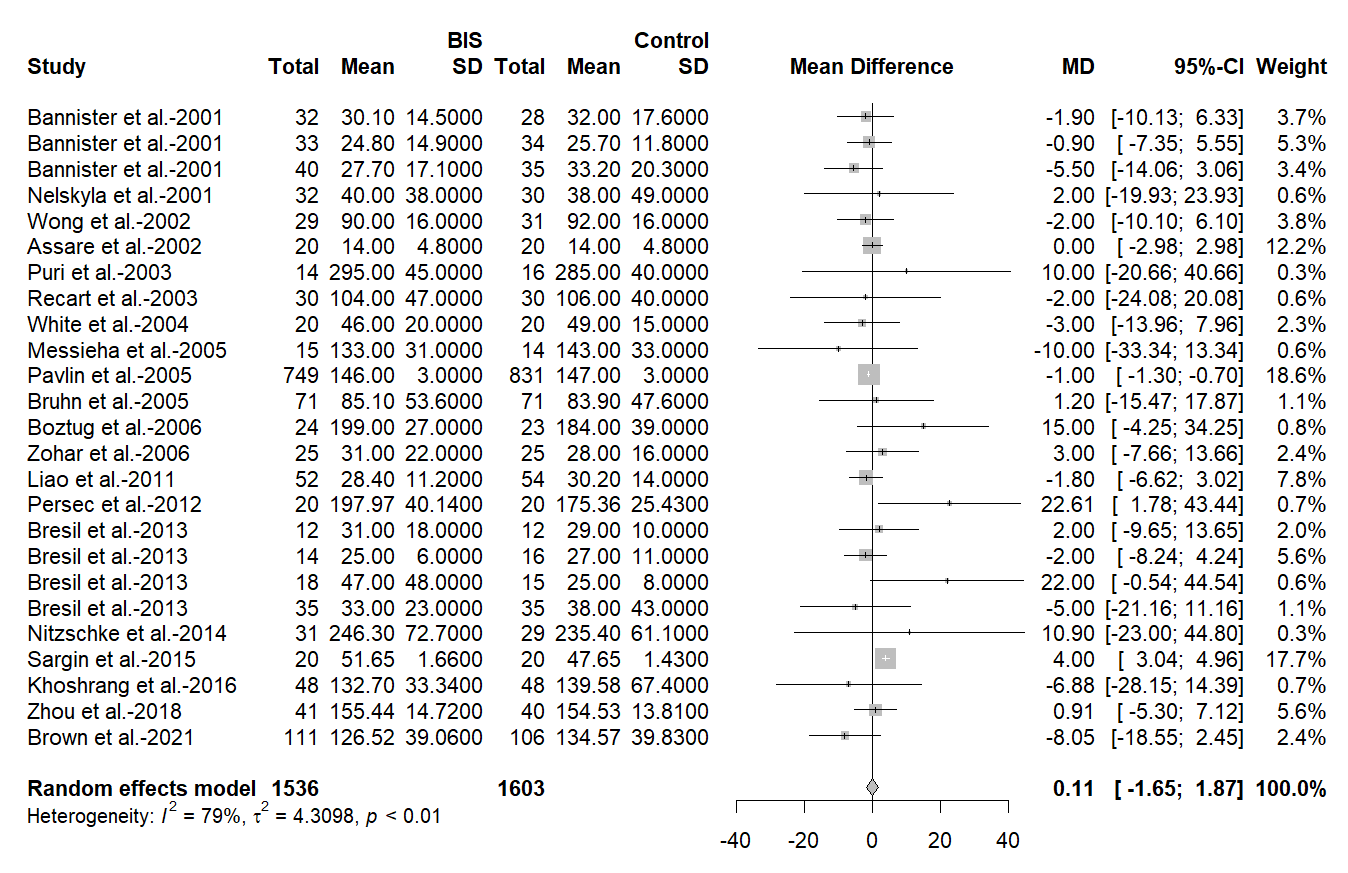


**Figure S11.** Meta-analysis results of surgery time


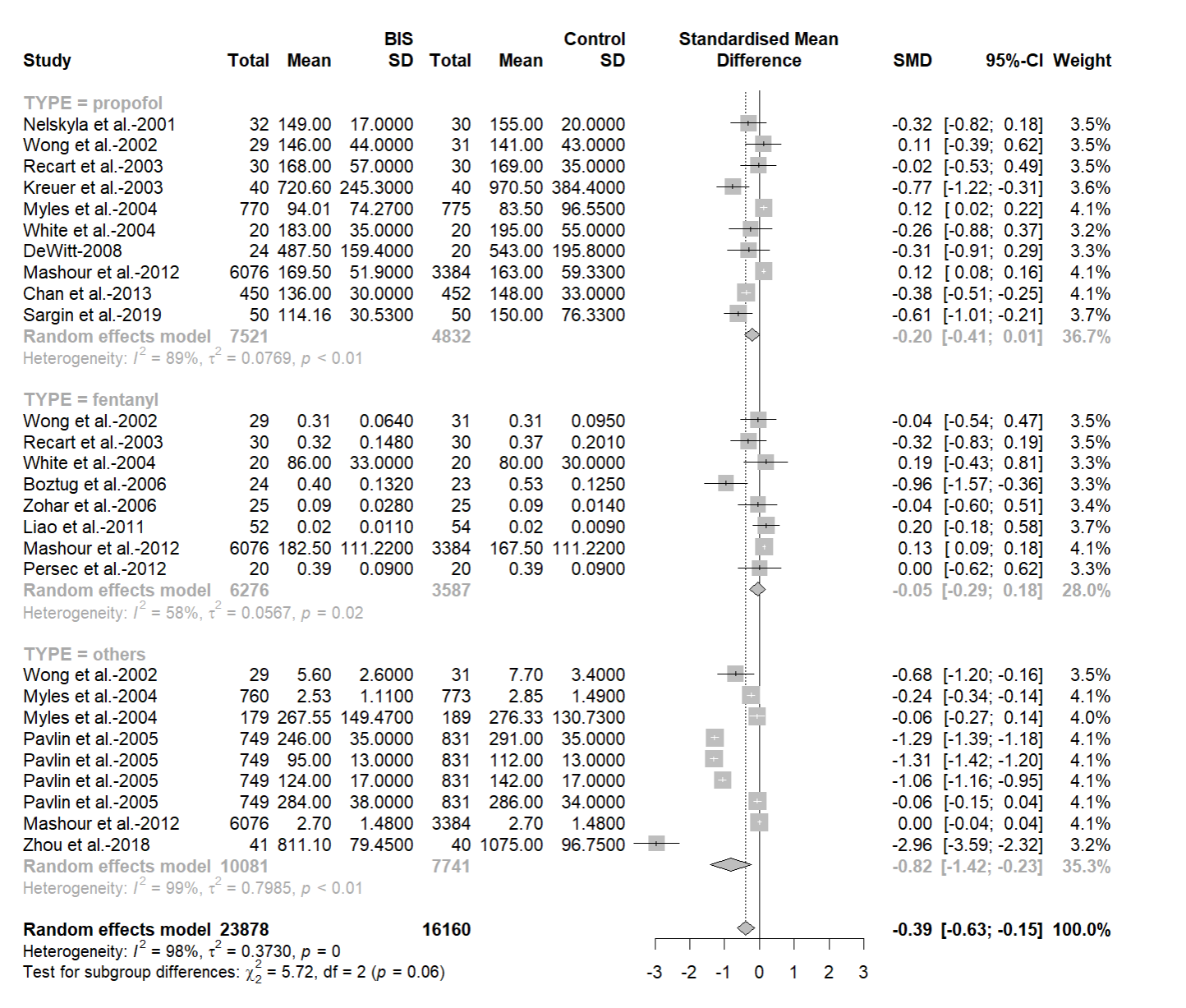

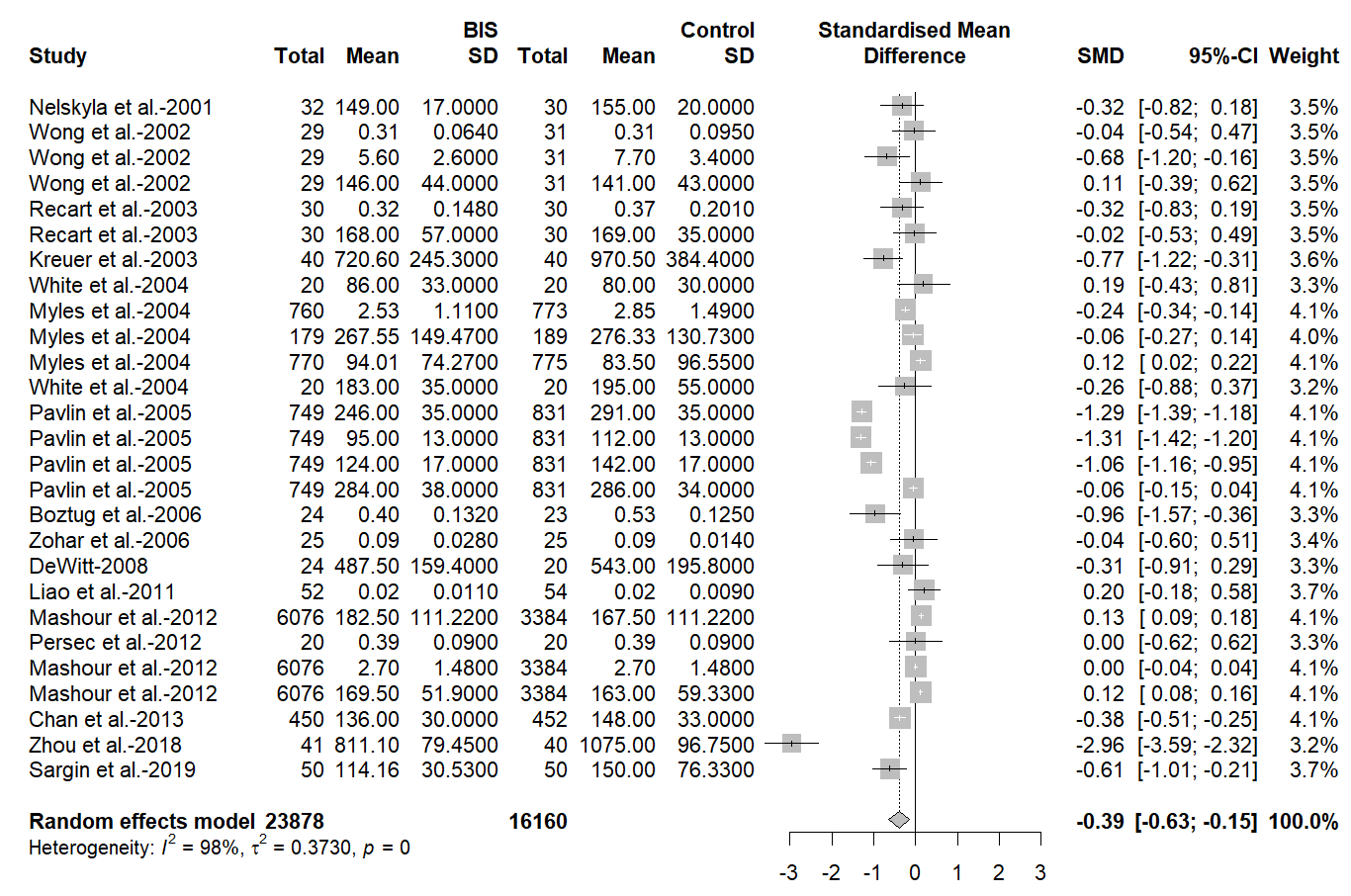
**Figure S12.** Meta-analysis results of anesthetic dosage

**Figure S13.** Subgroup meta-analysis results of anesthetic dosage

Supplementary 2.2 Funnel Plots


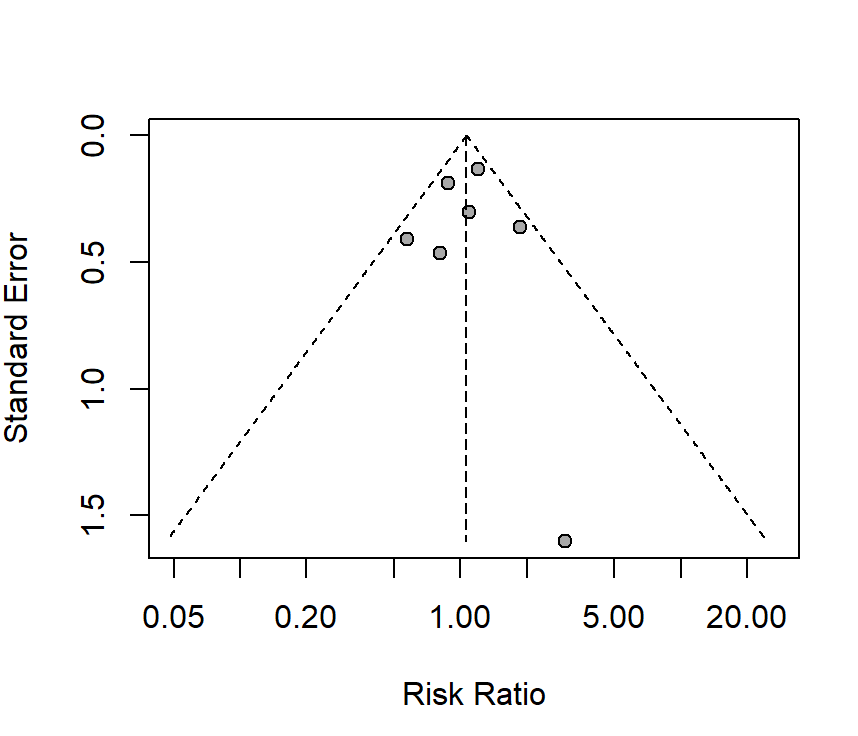

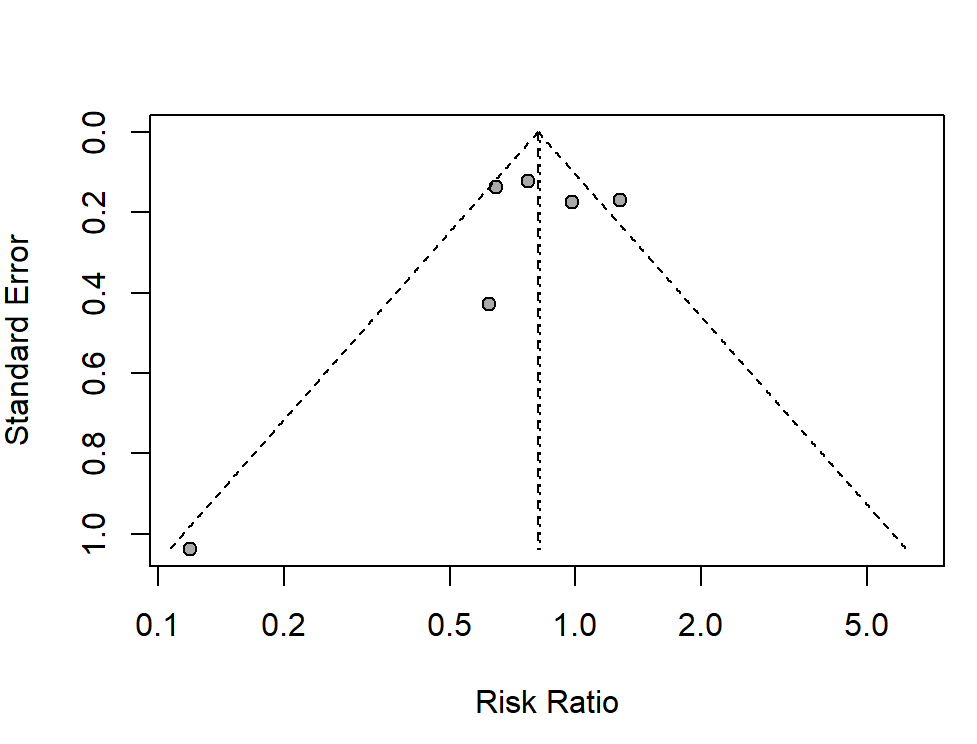
(1) Postoperative delirium (2) Postoperative nausea and vomiting (3) Abnormal blood pressure


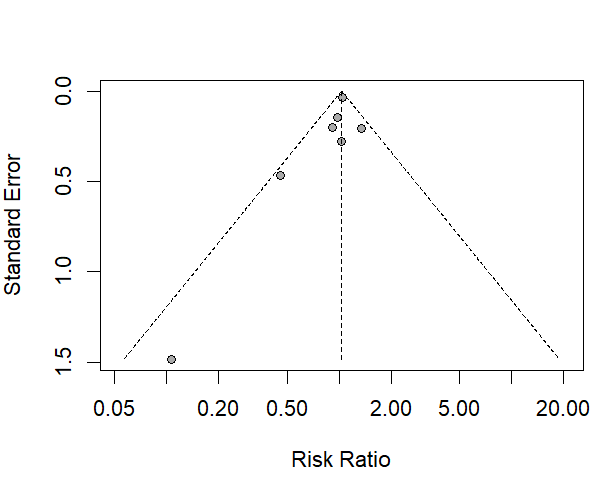


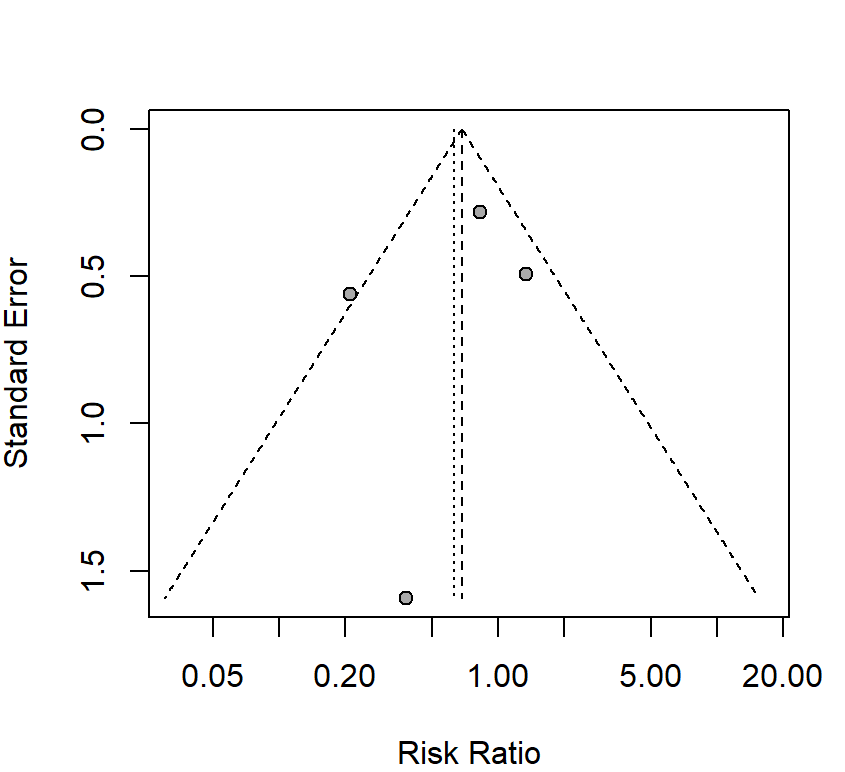
(4) Intraoperative awareness (5) Postoperative cognitive dysfunction (6) Mortality


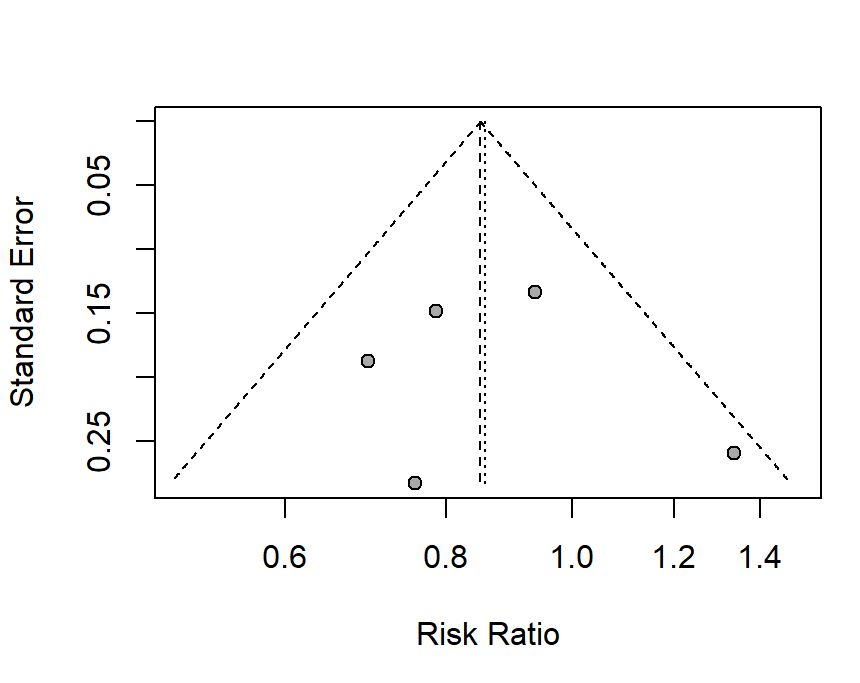

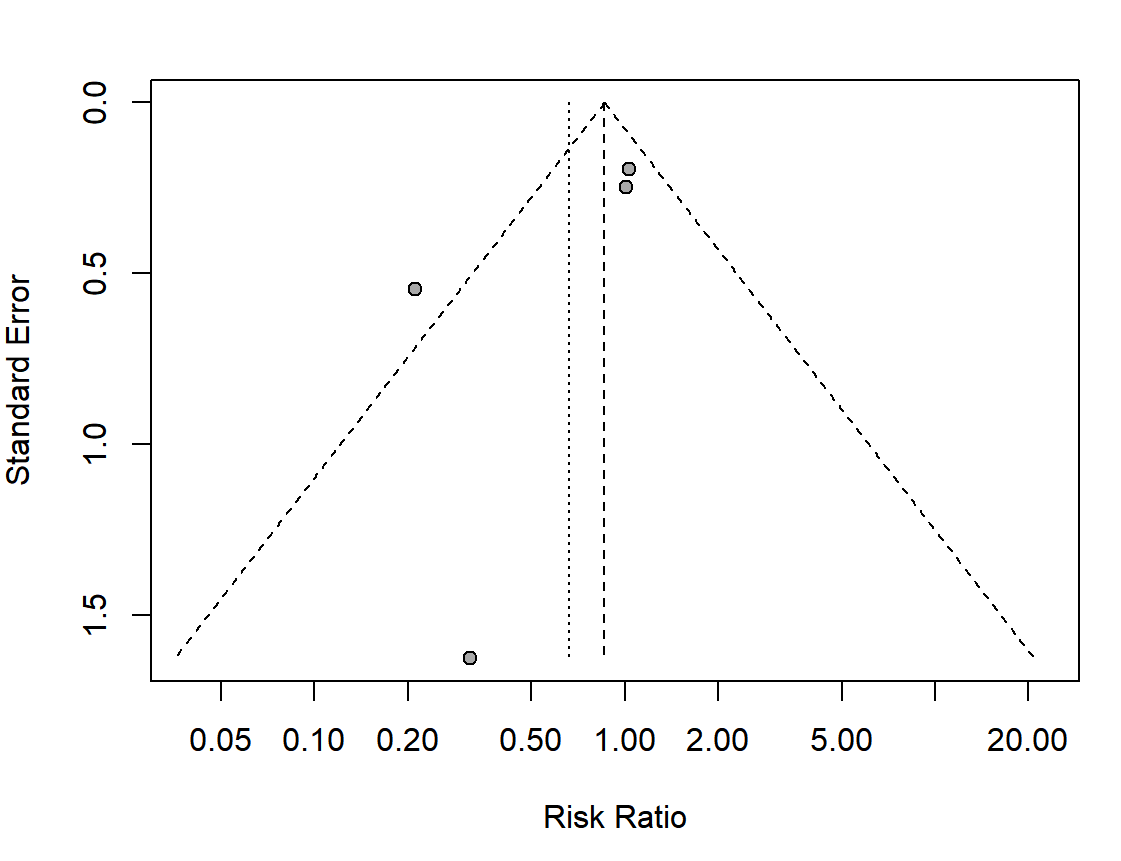


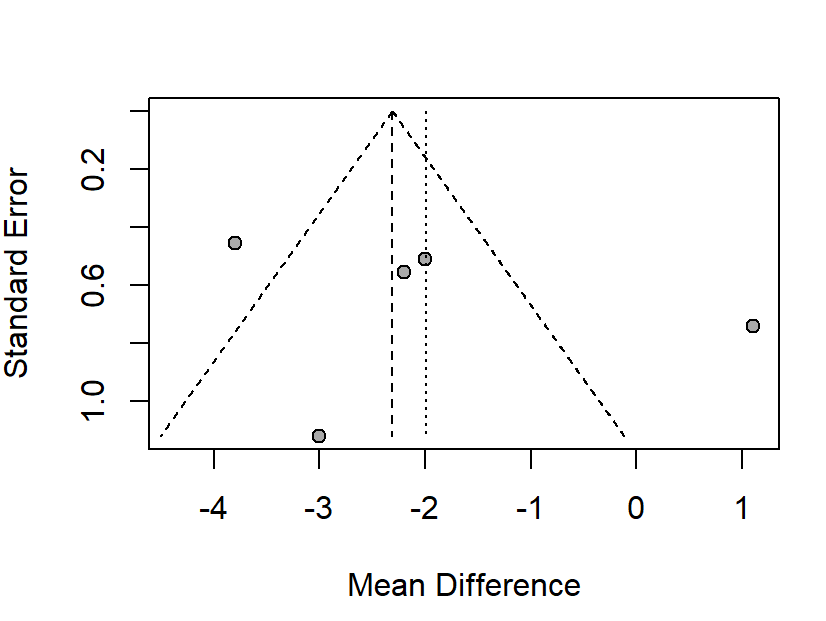
(7) Eye opening time (8) Orientation force recovery time (9) Extubation time


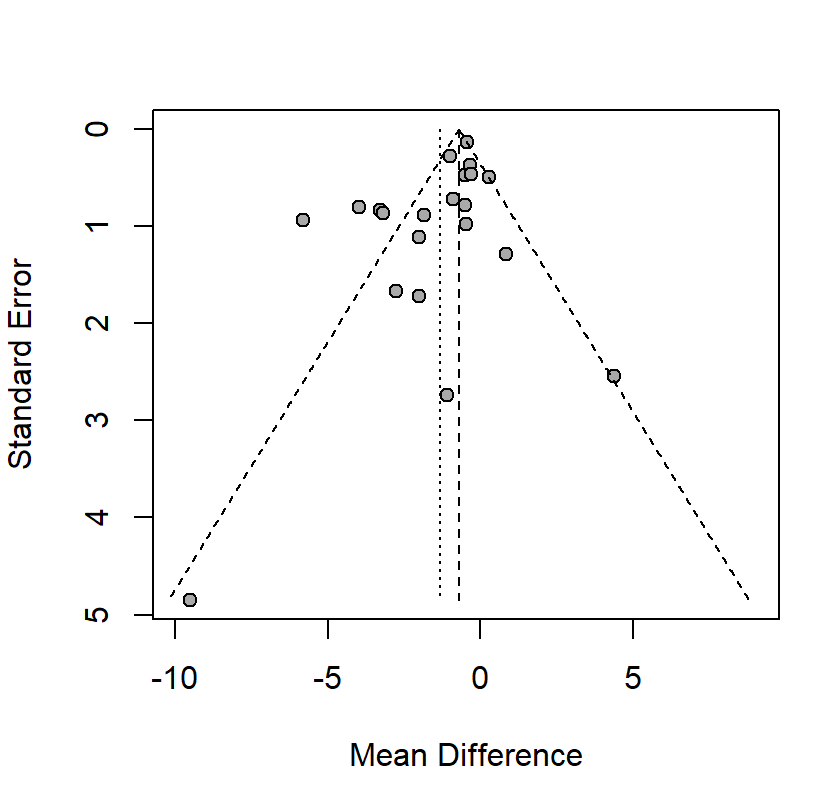

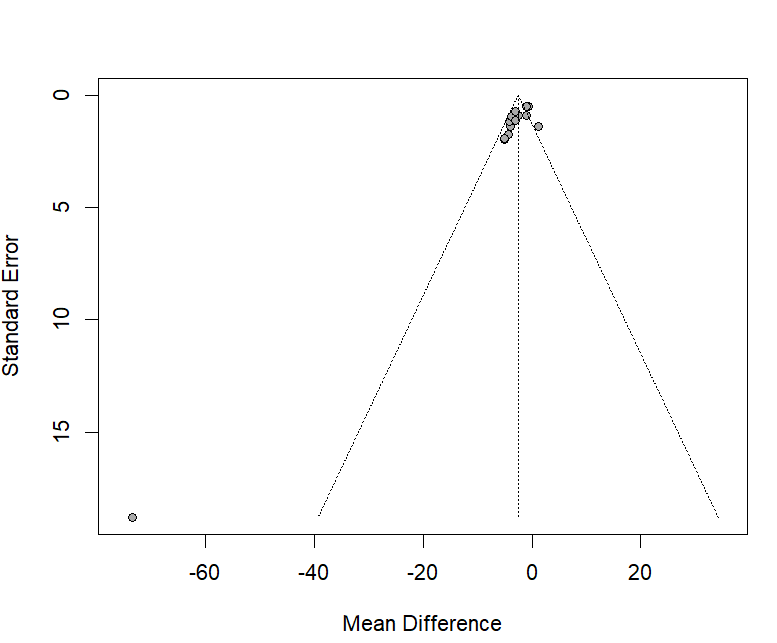


(10) PACU stay duration (11) Surgery time (12) Anesthetic dosage


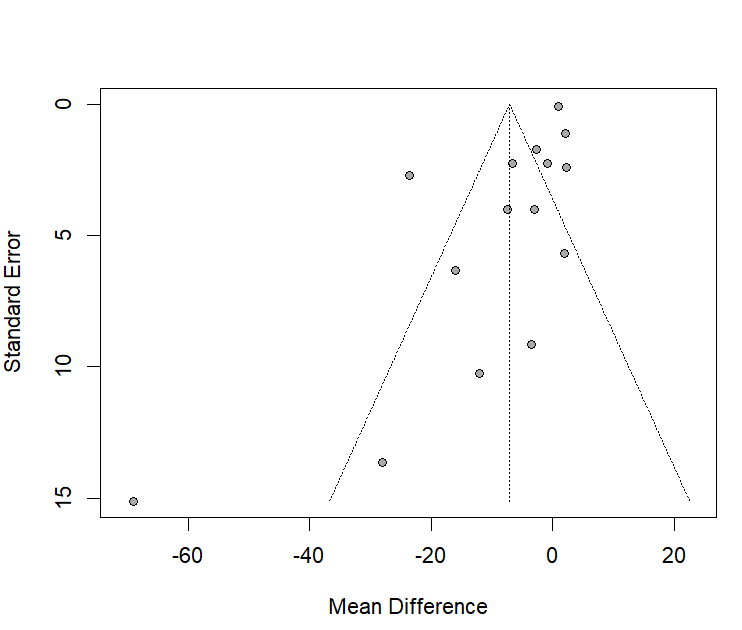


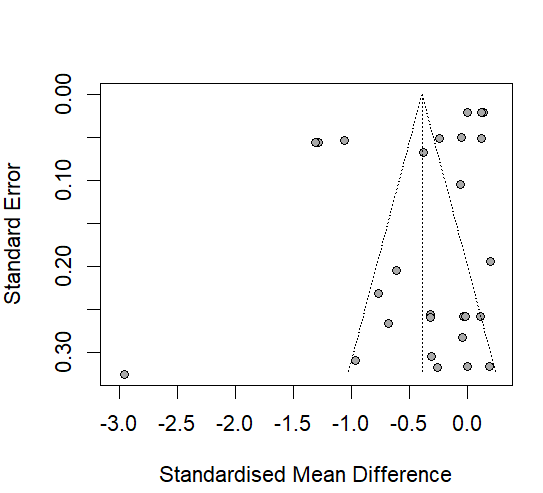

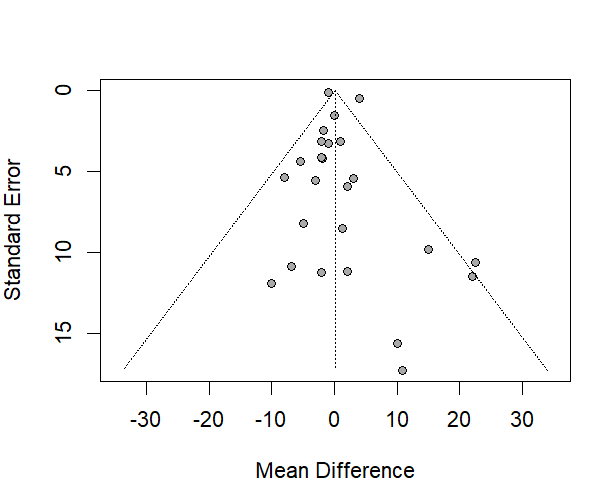

Supplement: Supplementary Materials — Supplementary 1: PRISMA 2020 Checklist; Supplementary 2: Supplementary 2.1: Forest Plots and Supplementary 2.2: Funnel Plots. Figure S1: meta-analysis results of postoperative delirium. Figure S2: meta-analysis results of postoperative nausea and vomiting. Figure S3: meta-analysis results of abnormal blood pressure. Figure S4: meta-analysis results of intraoperative awareness. Figure S5: meta-analysis results of postoperative cognitive dysfunction. Figure S6: meta-analysis results of mortality. Figure S7: meta-analysis results of eye-opening time. Figure S8: meta-analysis results of orientation force recovery time. Figure S9: meta-analysis results of extubation time. Figure S10: meta-analysis results of PACU stay duration. Figure S11: meta-analysis results of surgery time. Figure S12: meta-analysis results of anesthetic dosage. Figure S13: subgroup meta-analysis results of anesthetic dosage. [file 5555481.f1.zip › 5555481 Supplementary 2 Forest Plots and Funnel Plots.docx]
